# Supplementary material for: Charge-loop current order and Z3 nematicity mediated by bond order fluctuations in kagome metals
Source: Nat Commun. 2023 Nov 29;14:7845. doi: 10.1038/s41467-023-42952-6 (PMC10687221; doi:10.1038/s41467-023-42952-6)
Supplement: Supplementary file 1 — Supplementary Information [file 41467_2023_42952_MOESM1_ESM.pdf]

[Supplementary Information]

Charge-loop current order and Z_3-nematicity mediated by bond order fluctuations in kagome metals

Rina Tazai[1], Youichi Yamakawa[2], and Hiroshi Kontani[2]

[1] *Yukawa Institute for Theoretical Physics, Kyoto University, Kyoto 606-8502, Japan*

[2] *Department of Physics, Nagoya University, Furo-cho, Nagoya 464-8602, Japan.*

Supplementary Note 1: Square-lattice kagome model

In this Supplementary Information, we set the hopping integrals $t = -0.5$ eV and $t' = 0$. Hereafter, the unit of energy is eV unless otherwise noted. In Ref. [1], the present authors found that the paramagnon-interference theory naturally explains the bond order (BO) on the basis of the kagome-lattice Hubbard model. The used lattice structure with the square unit cell and its Fermi surface (FS) are shown in Supplementary Figs. 1 **a** and **b**, respectively. The van-Hove singular point at $\mathbf{k} = \mathbf{k}_X$ (X=A,B,C) is composed of the X-sublattice. The form factor of the $3Q$ BO and that of the $3Q$ cLC in real space are shown in Supplementary Figs. 1 **c** and **d**, respectively. We stress that the D_{6h} point group symmetry of the original kagome lattice is not harmed in the present DW equation solution using this square-lattice model.

Supplementary Note 2: BO fluctuations due to Fock term by V

Here, we discuss the important effect of the nearest-site Coulomb interaction V in addition to the on-site one U.

Supplementary Note 2-1: HF approximation for the U-V Hubbard model

The analysis of the kagome-lattice U-V Hubbard model based on the mean-field theory is presented in Section SF of Ref. [1]. The charge (spin) channel eigenvalue $\lambda^{c(s)}$ in the mean-field theory is given by solving the linearized DW equation with the Hartree-Fock (HF) kernel function made of U and V. Its diagrammatic expression for V is shown in Supplementary Fig. 2 **a**.

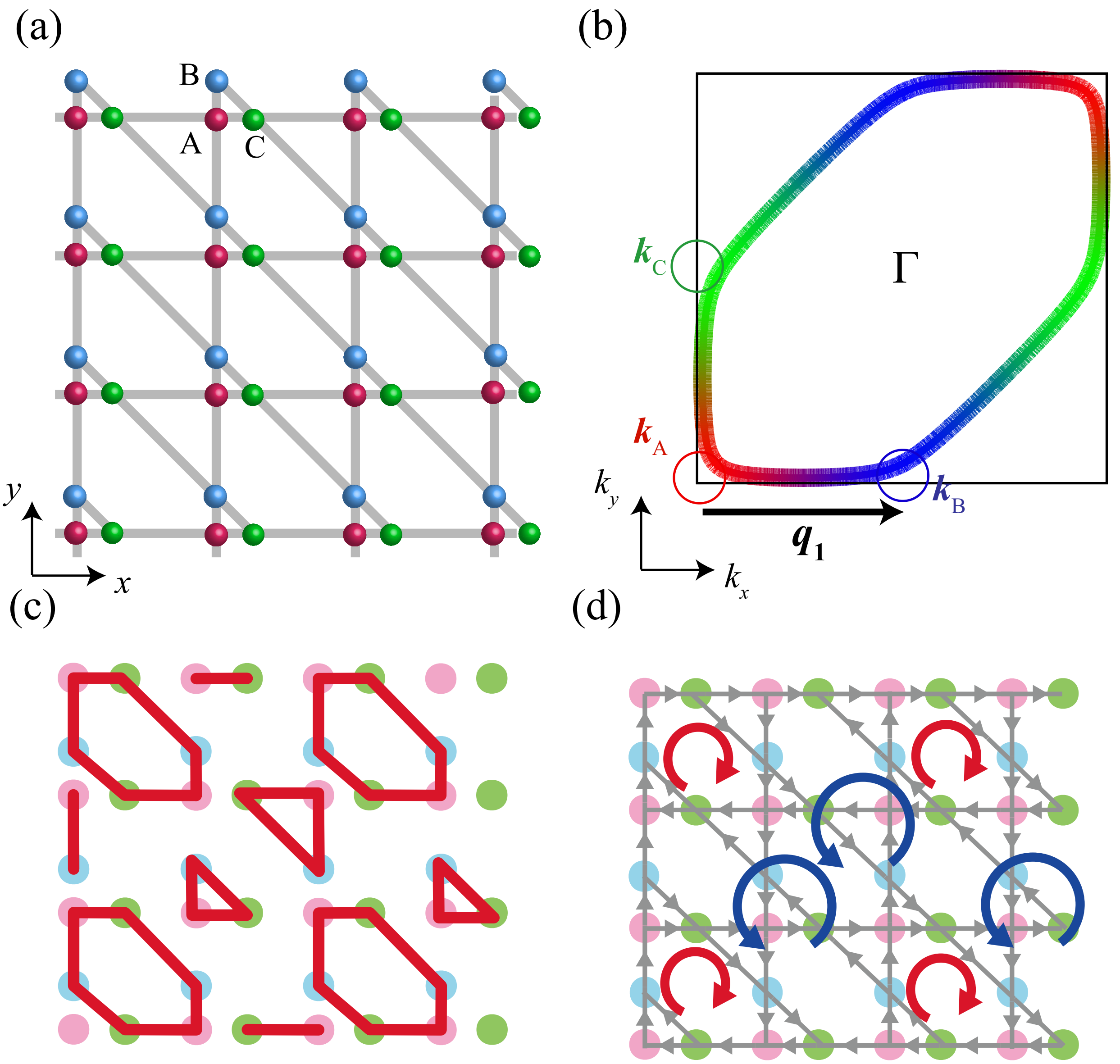

Supplementary Fig. 1: **a**. Square-lattice kagome metal model that is convenient for the numerical study. **b**. Obtained Fermi surface (FS) in the square Brillouin zone. The color represents the weight of the sublattice (A = red, B = blue, C = green). **c**. Form factor of the 3Q bond order (BO) in real space. The red bonds represent the Tri-Hexagonal pattern. **d**. Form factor of the 3Q charge loop current (cLC). The coexistence of **c** and **d** leads to the C_6 symmetry state.

Supplementary Figure 2 **b** shows the obtained several largest eigenvalues, $\lambda^s_{\rm SDW}$ and λ^c_X (X=CDW, BO, cLC), as functions of V/U at $U = 0.79$ [1]. These eigenvalues linearly increase with respect to U and V at a fixed V/U. When $V/U \ll 1$, a simple SDW order ($f = 1$) at $\mathbf{q} \approx \mathbf{0}$ is realized for $U \sim 1.6$. It originates from the Hartree term of U. When

$V/U \gg 1$, on the other hand, simple charge-density-wave (CDW) order ($f = 1$) at $\mathbf{q} \approx \mathbf{q}_n$ is caused by the Hartree term of V. In this model, $\mathbf{q}_1 = (\pi, 0)$, $\mathbf{q}_2 = (\pi, \pi)$, $\mathbf{q}_3 = (0, \pi)$. For $V/U = 0.4 \sim 0.65$, the BO is realized by the Fock term of V. Note that the non-local BO is not suppressed by U, while the simple CDW order due to the Hartree term of V is strongly suppressed by U. However, the cLC instability is smaller than other instabilities within the HF approximation.

The form factors of the BO between the nearest sites in the square kagome-lattice model in Supplementary Fig. 1 **a** are given by

$$b_{\mathrm{AB}}(\mathbf{q}) = (1 - e^{-iq_y})/2, \tag{1}$$

$$b_{\mathrm{BC}}(\mathbf{q}) = (1 - e^{-iq_x + iq_y})/2, \tag{2}$$

$$b_{\mathrm{CA}}(\mathbf{q}) = (1 - e^{iq_x})/2, \tag{3}$$

which are normalized as $\max_{\mathbf{q}}\{b_{lm}(\mathbf{q})\} = 1$. Here, $b_{lm}(\mathbf{q}) = b_{ml}(\mathbf{q})^*$ and $b_{ll}(\mathbf{q}) = 0$.

Supplementary Note 2-2: Effective interaction due to BO susceptibility

In Supplementary Fig. 2 **b**, we found the development of the BO instability within the mean-field approximation. Next, we derive the effective interaction mediated by the BO susceptibility. The final result is given in Supplementary Eq. (16), which is essentially equivalent to Eq. (10) derived in the main text.

From now on, we derive Supplementary Eq. (16). First, we consider the effective interaction due to the Fock term of V. The Hamiltonian is $H_V = \sum_{il,jm,\sigma\sigma'} V_{il,jm} c^\dagger_{il,\sigma} c_{il,\sigma} c^\dagger_{jm,\sigma'} c_{jm,\sigma'}$, where i, j represent the unit cell, $l, m = A, B, C$, and c_{il} is the electron annihilation operator. (Here, we drop the spin indices for simplicity.) H_V is rewritten as $H_V = \frac{1}{N}\sum_{\mathbf{k}\mathbf{k}'\mathbf{q}\sigma\sigma'} V_{lm}(\mathbf{q}) c^\dagger_{\mathbf{k}+\mathbf{q},l,\sigma} c_{\mathbf{k},l,\sigma} c^\dagger_{\mathbf{k}',m,\sigma'} c_{\mathbf{k}'+\mathbf{q},m,\sigma'}$. Here, $V_{lm}(\mathbf{q}) = \frac{1}{N}\sum_{i,j} V_{il,jm} e^{-i\mathbf{q}\cdot(\mathbf{r}_{i,l} - \mathbf{r}_{j,m})}$, where $\mathbf{r}_{i,l}$ is the coordinate of the site (i, l); In Supplementary Fig. 1 **a**, $\mathbf{r}_{i,l} = (i_x, i_y)$ is independent of l, and i_x, i_y are integer coordinates. In the case of the nearest-site Coulomb interaction V, $V_{lm}(\mathbf{q})$ is expressed as

$$V_{lm}(\mathbf{q}) = 2V a_{lm}(\mathbf{q}). \tag{4}$$

Here,

$$a_{\mathrm{AB}}(\mathbf{q}) = (1 + e^{-iq_y})/2, \tag{5}$$

$$a_{\mathrm{BC}}(\mathbf{q}) \;=\; (1+e^{-iq_x+iq_y})/2, \tag{6}$$

$$a_{\mathrm{CA}}(\mathbf{q}) \;=\; (1+e^{iq_x})/2, \tag{7}$$

where $a_{lm}(\mathbf{q}) = a_{ml}(\mathbf{q})^*$ and $a_{ll}(\mathbf{q}) = 0$.

Considering the relation $a_{lm}(\mathbf{k}-\mathbf{k}') = a_{lm}(\mathbf{k})a_{lm}(\mathbf{k}')^* + b_{lm}(\mathbf{k})b_{lm}(\mathbf{k}')^*$, the Fock term $V_{lm}(\mathbf{k}-\mathbf{k}')$ in Supplementary Fig. 2 **c** is expressed as

$$\begin{aligned} V_{lm}(\mathbf{k}-\mathbf{k}') &= 2V[a_{lm}(\mathbf{k})a_{lm}(\mathbf{k}')^* + b_{lm}(\mathbf{k})b_{lm}(\mathbf{k}')^*] \\ &= 2V\sum_{d}^{a,b} d_{lm}(\mathbf{k})d_{lm}(\mathbf{k}')^* \end{aligned} \tag{8}$$

which is expressed in Supplementary Fig. 2 **c**. Note that $a_{\mathrm{AB}}(\mathbf{k}) \approx a_{\mathrm{AB}}(\mathbf{k}') \approx 0$ and $b_{\mathrm{AB}}(\mathbf{k}) \approx b_{\mathrm{AB}}(\mathbf{k}') \approx 1$ for $(l,m) = (\mathrm{A},\mathrm{B})$ and $(\mathbf{k},\mathbf{k}') \approx (\mathbf{k}_{\mathrm{A}},\mathbf{k}_{\mathrm{B}})$. Because $a_{lm}(\mathbf{k})$ and $b_{lm}(\mathbf{k})$ are orthogonal, the form factor $g_{\mathbf{q}}^{lm}(\mathbf{k})$ for the largest eigenvalue of the DW equation is equal to $b_{lm}(\mathbf{k})$ within the Fock approximation.

We next consider the second-order term with respect to V shown in Supplementary Fig. 2 **c**:

$$\begin{aligned} V^{(2)}_{lm,l'm'}(\mathbf{k}-\mathbf{k}';\mathbf{q}) &= \frac{T}{N}\sum_{p} V_{lm}(\mathbf{k}-\mathbf{p})G_{ll'}(p+q)G_{m'm}(p)V_{l'm'}(\mathbf{p}-\mathbf{k}') \\ &= (2V)^2\sum_{d,d'}^{a,b} d_{lm}(\mathbf{k})(d'_{l'm'}(\mathbf{k}'))^*\chi^{0dd'}_{lm,l'm'}(q), \end{aligned} \tag{9}$$

where $\chi^{0dd'}_{lm,l'm'}(q) = -\frac{T}{N}\sum_p (d_{lm}(\mathbf{p}))^* G_{ll'}(p+q)G_{m'm}(p)d'_{l'm'}(\mathbf{p})$ $(d,d' = a,b)$. In kagome metal model, $\chi^{0dd'}_{lm,l'm'}(q)$ takes large value only for $d = d'$ and $\mathbf{q} \sim \mathbf{q}_m$ $(m = 1,2,3)$.

As we mentioned above, we obtain $a_{\mathrm{AB}}(\mathbf{k}) \approx a_{\mathrm{AB}}(\mathbf{k}') \approx 0$ and $b_{\mathrm{AB}}(\mathbf{k}) \approx b_{\mathrm{AB}}(\mathbf{k}') \approx 1$ for $(\mathbf{k},\mathbf{k}') \approx (\mathbf{k}_{\mathrm{A}},\mathbf{k}_{\mathrm{B}})$. By considering these relations, we can drop a_{lm} in Supplementary Eqs. (8) and (9). Thus, the summation of the first and the second order terms is given as

$$2Vb_{lm}(\mathbf{k})b_{l'm'}(\mathbf{k}')^*[\hat{1} + 2V\hat{\chi}^{0b}(q)]_{lm,l'm'}, \tag{10}$$

where $\chi^{0b}_{lm,l'm'}(q) \equiv \chi^{0bb}_{lm,l'm'}(q)$.

Now, we consider all the ladder diagrams composed of the Fock terms shown in Supplementary Fig. 2 **d**. It is obtained as

$$W_{lm,l'm'}(k,k',q) \approx 2Vb_{lm}(\mathbf{k})b_{l'm'}(\mathbf{k}')^*[\hat{1} + 2V\hat{\chi}^{b}(q)]_{lm,l'm'}, \tag{11}$$

which takes sizable value when $\mathbf{k} \approx \mathbf{k}_m$, $\mathbf{k}' \approx \mathbf{k}_{m'}$, and $\mathbf{q} \approx (\mathbf{k}_l - \mathbf{k}_m)$, $(\mathbf{k}_{l'} - \mathbf{k}_{m'})$ (modulo original reciprocal vectors). The BO susceptibility in Supplementary Eq. (11) is given by the solution of $\hat{\chi}^b(q) = \hat{\chi}^{0b}(q) + (2V)\hat{\chi}^{0b}(q)\hat{\chi}^b(q)$. It is expressed as

$$\hat{\chi}^b(q) = \hat{\chi}^{0b}(q)(\hat{1} - 2V\hat{\chi}^{0b}(q))^{-1}. \tag{12}$$

Thus, $\hat{W}(k,k',q)$ is proportional to the BO susceptibility $\hat{\chi}^b(q)$. (One can verify the relation $\hat{\chi}^b(q) = \hat{\chi}^{0b}(q) + \frac{T^2}{N^2}\sum_{kk'} \hat{A}(k,q)\hat{W}(k,k',q)\hat{A}(k',q)$, where $A_{ml,m'l'}(k,q) = G_{mm'}(k+q)G_{l'l}(k)$.)

By using Supplementary Eq. (11), the charge-channel MT term in the DW equation is

$$I_{ll'mm'}(k,k',q) = -2W_{m'l'ml}(k,k+q,k'-k), \tag{13}$$

where the factor 2 comes from the summation of the parallel-spin $(\uparrow,\uparrow)$ and the antiparallel-spin $(\uparrow,\downarrow)$ ladder diagrams, because the Pauli principle does not work on V. It is expressed in Supplementary Fig. 2 **d**. Note that the present Fock term of the off-site Coulomb interaction corresponds to the Hartree term of the e-ph interaction in Eq. (2) in the main text.

As discussed above, $\chi^{0b}_{lm,l'm'}(q)$ is enlarged at $\mathbf{q} \approx \mathbf{q}_1$ only when $(lm,l'm') = (\mathrm{AB},\mathrm{AB}),(\mathrm{AB},\mathrm{BA})$. (In the same way, it is enlarged at $\mathbf{q} \approx \mathbf{q}_2$ only when $(lm,l'm') = (\mathrm{BC},\mathrm{BC}),(\mathrm{BC},\mathrm{CB})$.) For this reason, we can safely approximate Supplementary Eqs. (11) and (12) as the 2×2 matrix expressions for $\mathbf{q} \approx \mathbf{q}_n$ $(n = 1,2,3)$.

Below, we explain that the relation $\chi^b_{\mathrm{AB,AB}}(\mathbf{q}_1) \approx \chi^b_{\mathrm{AB,BA}}(\mathbf{q}_1)$ holds. In kagome metals, the ratio in the irreducible susceptibility $\chi^{0b}_{\mathrm{AB,BA}}(\mathbf{q}_1)/\chi^{0b}_{\mathrm{AB,AB}}(\mathbf{q}_1)$ is just ~ 0.2 because the Green function $G_{lm}(k)$ is nearly diagonal ($\propto \delta_{l,m}$). Nonetheless of this fact, the ratio in the BO susceptibility $R \equiv \chi^b_{\mathrm{AB,BA}}(\mathbf{q}_1)/\chi^b_{\mathrm{AB,AB}}(\mathbf{q}_1)$ is of order unity, when the BO Stoner factor $\alpha_{\mathrm{BO}} = 2V(\chi^{0b}_{\mathrm{AB,AB}}(\mathbf{q}_1) + \chi^{0b}_{\mathrm{AB,BA}}(\mathbf{q}_1))$ is close to unity. In fact, for $\mathbf{q} \approx \mathbf{q}_1$, the relation between the BO susceptibility and its irreducible susceptibility is $\hat{\chi}^b = \hat{\chi}^{b0} + 2V\hat{\chi}^{b0}\hat{\chi}^b$, where $\hat{\chi}^{b0}$ is the 2×2 matrix: $\hat{\chi}^{b0} = \begin{pmatrix} \chi^{b0}_{\mathrm{AB,AB}} & \chi^{b0}_{\mathrm{AB,BA}} \\ \chi^{b0}_{\mathrm{BA,AB}} & \chi^{b0}_{\mathrm{BA,BA}} \end{pmatrix}$. Then, the BO susceptibility for $\mathbf{q} \approx \mathbf{q}_1$ is obtained as

$$\chi^b_{\mathrm{AB,AB}}(q) = [a - 2V(a^2 - b^2)]/d, \tag{14}$$

$$\chi^b_{\mathrm{AB,BA}}(q) = b/d, \tag{15}$$

where $a \equiv \chi^{0b}_{\mathrm{AB,AB}}(q)$, $b \equiv \chi^{0b}_{\mathrm{AB,BA}}(q)$, and $d \equiv (1 - (a+b)2V)(1 - (a-b)2V)$.

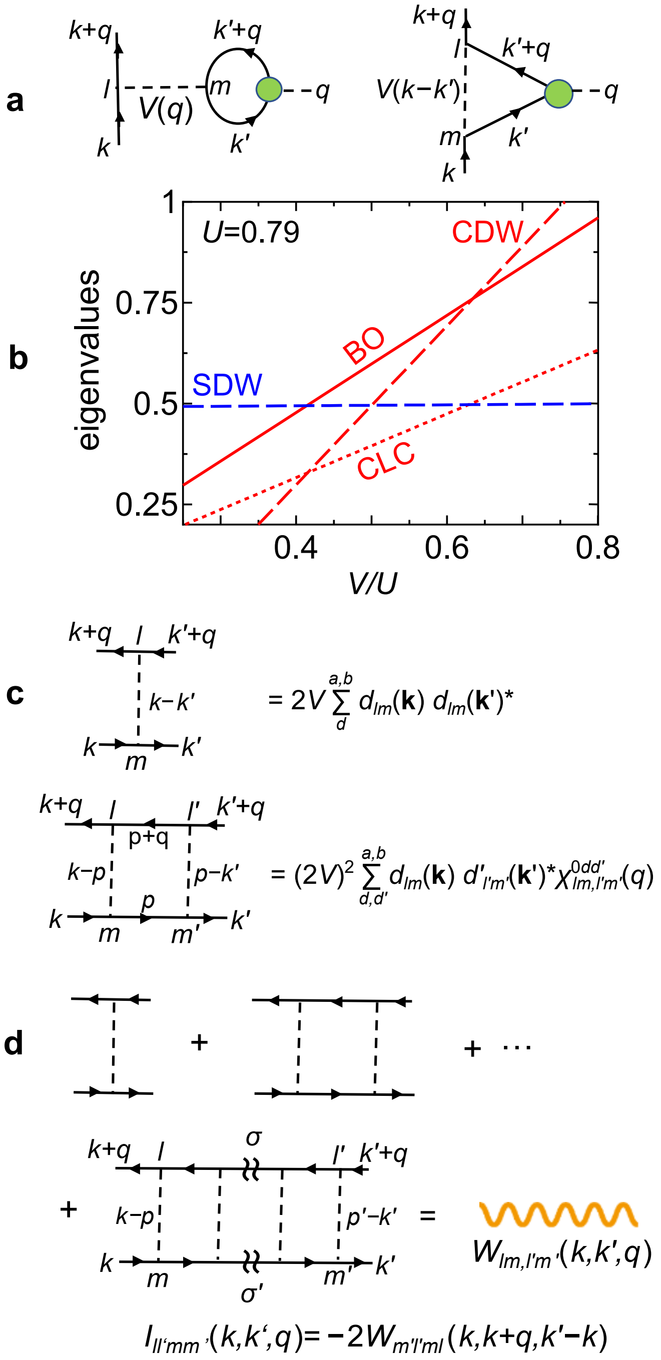

Supplementary Fig. 2: **a**. Hartree and Fock terms with respect to the on-site (U) and the nearest-site (V) Coulomb interactions in the density-wave (DW) equation. Each green circle represents the form factor. **b**. Eigenvalues of the HF DW equation as a function of V/U: $\lambda^s_{\rm SDW}$ and λ^c_X (X=CDW, BO, cLC). Note that $\lambda^c_{\rm BO} \approx \lambda^s_{\rm BO}$; see Ref. [1]. **c**. The first- and the second-order terms with respect to the Fock terms of V. **d** W given by the summation of all ladder-type diagrams, which is proportional to the BO susceptibility. The present Fock term corresponds to the Hartree term of the e-ph interaction in Eq. (2) in the main text.

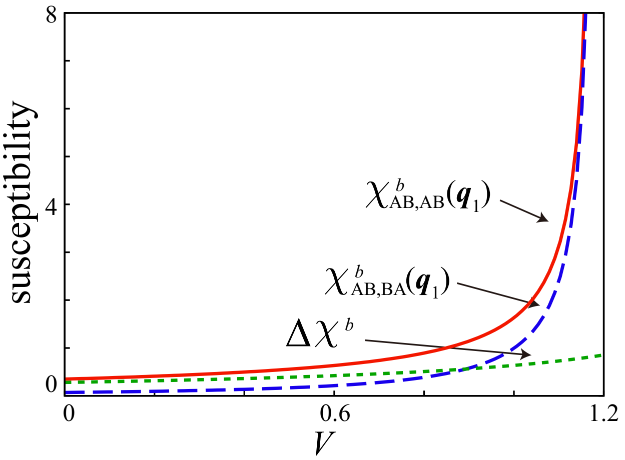

Supplementary Fig. 3: $\chi^b_{\rm AB,AB}$ and $\chi^b_{\rm AB,BA}$ given in Supplementary Eqs. (14) and (15) as functions of V. We set $a = 0.35$ and $b/a = 0.2$. $\Delta\chi^b \equiv \chi^b_{\rm AB,AB} - \chi^b_{\rm AB,BA}$ is also shown.

According to Supplementary Eqs. (14) and (15), R becomes $b/a(\ll 1)$ when $V = 0$. In contrast, we obtain $R \approx 1$ at $\alpha_{\rm BO} \approx 1$ ($\alpha_{\rm BO} = (a+b)2V$). Supplementary Figure 3 shows the BO susceptibilities $\chi^b_{\rm AB,AB}$ and $\chi^b_{\rm AB,BA}$ as functions of V. We set $a = \chi^{b0}_{\rm AB,AB}(\mathbf{q}_1) = 0.35$ and $b/a = 0.2$. Both $\chi^b_{\rm AB,AB}$ and $\chi^b_{\rm AB,BA}$ increase with V, while $\Delta\chi^b \equiv \chi^b_{\rm AB,AB} - \chi^b_{\rm AB,BA}$ is almost constant. This result means that the relation $R \approx 1$ holds near the BO-endpoint, around which the relation $\alpha_{\rm BO} \lesssim 1$ holds.

According to Supplementary Eqs. (11) and (13), in the case of $R \approx 1$, the MT kernel function for the DW equation is simply given as

$$I_{ll',mm'}(k,k',q) \approx -g^{m'l'}_{k'-k}(k) g^{lm}_{k-k'}(k'+q) \times 2(2V)^2 \chi^b(k-k'), \quad (16)$$

where $\chi^b(q) \equiv \chi^b_{\rm AB,AB}(q) + \chi^b_{\rm AB,BA}(q) \approx \chi^{b0}_{\rm AB,AB}(q)/(1-\alpha_{\rm BO}(q))$ for $\mathbf{q} \approx \mathbf{q}_1$, and $g^{lm} \approx b_{lm}$ is the normalized BO form factor derived from the DW equation [1]. Supplementary Equation (16) is equal to Eq. (10) in the main text with $y = 2$. Note that both charge- and three spin-channel BO fluctuations develop in the present off-site V mechanism.

To summarize, the relation $\chi^b_{\rm AB,AB} \approx \chi^b_{\rm AB,BA}$ (*i.e.*, $R \approx 1$), which is assumed in the cLC mechanism in the main text, is well satisfied in the off-site V mechanism. Because this relation is also satisfied in the paramagnon-interference mechanism [1] and the phonon mechanism, these three different BO mechanisms will cooperate. We consider that the main mechanism of the BO in kagome metals is the paramagnon interference mechanism [1], and

both the bond-stretching phonon mode and the off-site Coulomb interaction will assist the BO formation.

Supplementary Note 2-3: The relation $y \gtrsim 1/2$ when v and V coexist

We consider the MT kernel function in the presence of both the off-site Coulomb interaction V and the BO interaction v in Eq. (2) in the main text. In this case, $y \gtrsim 1/2$ is expected to be realized. Considering the $SU(2)$ symmetry in spin space, the spin indices of the MT term in Supplementary Eq. (16) are written as [45]

$$
\begin{aligned}
I^{\sigma\sigma',\rho\rho'}_{ll',mm'}(k,k',q) &= \frac{1}{2} I^{\mathrm{c}}_{ll',mm'}(k,k',q)\delta_{\sigma,\sigma'}\delta_{\rho',\rho} \\
&\quad + \frac{1}{2} I^{\mathrm{s}}_{ll',mm'}(k,k',q)\boldsymbol{\sigma}_{\sigma,\sigma'}\cdot\boldsymbol{\sigma}_{\rho',\rho},
\end{aligned}
\tag{17}
$$

where $\boldsymbol{\sigma}$ is the Pauli matrix vector. $\hat{I}^{\mathrm{s(c)}}$ is the spin (charge) channel interaction given as $I^{\mathrm{s(c)}}_{ll'mm'} = -g^{m'l'}_{k'-k}(k) g^{lm}_{k-k'}(k'+q)(2V)^2 \chi^{b,\mathrm{s(c)}}(k-k')$, where $\chi^{b,\mathrm{s(c)}}(q) \approx \dfrac{\chi^{b0}_{\mathrm{AB,AB}}(q)}{1-\alpha^{\mathrm{s(c)}}(q)}$, and $\alpha^{\mathrm{s(c)}}(q)$ is the spin (charge) channel Stoner factor. Based on Supplementary Eq. (17), the MT kernel function for the charge-channel form factor is given as $\hat{I}^{\mathrm{MT}} = \frac{1}{2}(\hat{I}^{\mathrm{c}} + 3\hat{I}^{\mathrm{s}})$ [45]. When $v = 0$, $\alpha_{\mathrm{s,c}}(q)$ is equal to $\alpha_{\mathrm{BO}}(q)$ and therefore $\hat{I}^{\mathrm{c}} = \hat{I}^{\mathrm{s}}$. As a result, $\hat{I}^{\mathrm{MT}} = 2\hat{I}^{\mathrm{c}}$ for $v = 0$, and therefore $y = 2$.

Here, we consider the effect of the BO interaction v. When $v > 0$, the charge Stoner factor is magnified as $\alpha_{\mathrm{c}}(q) = \alpha_{\mathrm{BO}}(q) + v\chi^0_g(\mathbf{q})$, where $\chi^0_g(\mathbf{q})$ is the irreducible susceptibility given by Eq. (5) in the main text. Thus, $\hat{I}^{\mathrm{c}}$ is enlarged by v. In contrast, the spin Stoner factor and $\hat{I}^{\mathrm{s}}$ are unchanged by v. Even if v is small but finite (*e.g.*, $v\chi^0_g(\mathbf{q}) \sim 0.1$), the relation $\hat{I}^{\mathrm{c}} \gg \hat{I}^{\mathrm{s}}$ will be realized near the charge-channel BO criticality. Therefore, we obtain $\hat{I}^{\mathrm{MT}} \approx \frac{1}{2}\hat{I}^{\mathrm{c}}$, which means that $y \gtrsim 1/2$. To summarize, the relation $y \gtrsim 1/2$ is generally expected when V and v (= AL interference mechanism and e-ph interaction) coexist.

Supplementary Note 3: Self-consistent DW equation method: derivation of renormalized BO fluctuations

In the main text, we discuss the development of the BO susceptibility χ_g and its essential role in the cLC ordar. Based on the BO interaction model in Eq. (2) in the main text, we find that the MT term due to χ_g, $I^{\mathrm{MT}} \sim -\chi_g$, leads to the emergence of the cLC order. On

the other hand, $I^{\rm MT}$ also induces the renormalization of the χ_g itself. Here, we explain that the latter effect does not change the results of the main text. The kernel function composed of the Hartree and MT terms, shown in Supplementary Fig. 4 **a**, are respectively given as

$$\tilde{I}^{ll',mm'}_{{\rm MT},\mathbf{q}}(k,p) = -g^{m'l'}_{\mathbf{p}-\mathbf{k}}(\mathbf{k})g^{lm}_{\mathbf{k}-\mathbf{p}}(\mathbf{p}+\mathbf{q}) \times y\tilde{v}(1+\tilde{v}\tilde{\chi}_g(k-p)), \quad (18)$$

$$I^{ll',mm'}_{{\rm H},\mathbf{q}}(k,p) = g^{ll'}_{\mathbf{q}}(k)vg^{mm'}_{\mathbf{q}}(p)^*, \quad (19)$$

where $\tilde{\chi}_g(q) = \chi^0_g(q)/(1-\tilde{v}\chi^0_g(q))$ is the renormalized BO susceptibility. In the main text, v in the DW equation for the cLC form factor and in the self-energy is considered as the renormalized $\tilde{v}$.

Here, by referring to the SCR theory [10], we perform the self-consistent calculation of the renormalized BO susceptibility, $\tilde{\chi}_g(q)$, based on the DW equation. A natural self-consistency condition for the MT term composed of BO susceptibility is

$$\lambda_{\mathbf{q}_1} = \tilde{\alpha}_{\rm BO}, \quad (20)$$

where the left-hand side is the eigenvalue of the BO type form factor, and $\tilde{\alpha}_{\rm BO} \equiv \tilde{v}\chi^0_g(\mathbf{q}_1)$ is the renormalized BO Stoner factor. Then, we can calculate the DW equation under the self-consistent condition of BO fluctuations. $\tilde{v}$ given by Supplementary Eq. (20) is expressed as

$$\tilde{v} = v + v', \quad (21)$$

$$v' = \frac{\frac{T^2}{N^2}\sum_{k,p,L,M} A^L_{g'}(k+\mathbf{q},-\mathbf{q})I^{L,M}_{{\rm MT},\mathbf{q}}(k,p)A^M_{g'}(p,\mathbf{q})}{(\chi^0_{g'}(\mathbf{q}))^2}, \quad (22)$$

$$\hat{A}_{g'}(p,\mathbf{q}) = \hat{G}(p+\mathbf{q})\hat{g}'_{\mathbf{q}}(p)\hat{G}(p), \quad (23)$$

at $\mathbf{q} = \mathbf{q}_1$, where g' is the solution of the BO type form factor. The kernel function $I_{\mathbf{q}}(k,p)$ is shown in Supplementary Fig. 4 **a**. It is composed of the Hartree and MT terms. (Fock term is included in the MT term.) Because $g' \approx g$, $\tilde{v}$ is simply derived from Supplementary Eq. (21) by setting $g' = g$. By using $\tilde{v}$, we solve the DW equation and show the obtained $\lambda_{\rm BO}(=\tilde{\alpha}_{\rm BO})$ and $\lambda_{\rm cLC}$ for $y = 1$ and $T = 0.01$ in Supplementary Fig. 4 **b**.

For small v ($\lesssim 0.2$), the DW equation solution in Supplementary Fig. 4 **b** corresponds to th HF approximation. When $f_{\mathbf{q}}(k)$ is BO form factor, the eigenvalue due to the Hartree term

is equal to $\alpha_{\rm BO} = 1.34v$. However, it is reduced to $\lambda_{\rm BO} = 1.34(v+v')$, where $v' = -0.31yv$ is the Fock term contribution. When $f_{\mathbf{q}}(k)$ is the cLC form factor, the Hartree term vanishes, while the Fock term gives positive eigenvalue $\lambda_{\rm cLC} = 1.34v''$ with $v'' = 0.26yv$. For large v ($\gtrsim 1$), the MT term becomes significant. For this reason, $\lambda_{\rm BO}$ saturates while $\lambda_{\rm cLC}$ strongly increases, and $\lambda_{\rm cLC}$ reaches unity at $v \approx 1.35$. This is because both $-v'$ and v'' are strongly enlarged by the MT term when $\tilde{\alpha}_{\rm BO} \lesssim 1$.

Supplementary Figures 4 **c** and **d** show the obtained $T_{\rm cLC}$ and $T_{\rm BO}$ by solving the present DW equation, in the case of **c** $y = 1$ and **d** $y = 0.5$. Here, $T_{\rm cLC}$ is defined by the condition $\lambda_{\mathbf{q}_1} = 1$ for the cLC type form factor. Also, $T_{\rm BO}$ is defined as $\lambda_{\mathbf{q}_1} = 0.985$ for the BO type form factor. The overall v-dependences of the order parameters are similar to those in Figs. 4 **d** and **e** in the main text. By introducing the self-energy, both $T_{\rm cLC}$ and $T_{\rm BO}$ will be suppressed, and the relation $T_{\rm cLC} < T_{\rm BO}$ will be realized in the strong coupling region.

Supplementary Figure 4 **e** presents the beyond-RPA processes $\Delta\chi_w(q)$ in the present study. The total susceptibility is $\chi_w^{\rm tot}(q) = \chi_w^{\rm RPA}(q) + \Delta\chi_w(q)$, All these diagrams are generated by solving the DW equation. For $w = g$ (=BO form factor), $\chi_g^{\rm RPA}(q)$ is large and positive, while $\Delta\chi_g(q)$ takes negative values due to the MT terms. For $w = f$ (=cLC form factor), $\chi_f^{\rm RPA}(q)$ is very small, while $\Delta\chi_f(q)$ takes positive values, which becomes significant when $\alpha_{\rm BO} \sim 1$. Therefore, the cLC susceptibility $\chi_f^{\rm tot}(q)$ develops as large as the BO susceptibility in the present theory.

In the main text, we solve the DW equation with including the self-energy, while we solve the DW equation under the self-consistent condition in Supplementary Eq. (20) in this section. We find that two different DW equation analyses with MT-type kernel function produce essentially equivalent numerical results. The present self-consistent DW equation analysis strongly supports the reliability of the numerical study in the main text.

Supplementary Note 4: Effects of AL-type VCs

Here, we examine the role of the Aslamazov-Larkin (AL) vertex corrections (VCs) due to the interference between two bosonic susceptibilities ($\chi^{\rm boson}$) shown in Supplementary Fig. 5. The AL terms are significant for the even-parity order parameter in the paramagnon-interference mechanism. This mechanism is responsible for the BO and the orbital order in Fe-based superconductors [2, 3, 9], high-T_c cuprates [5, 6, 11], and kagome metals [1].

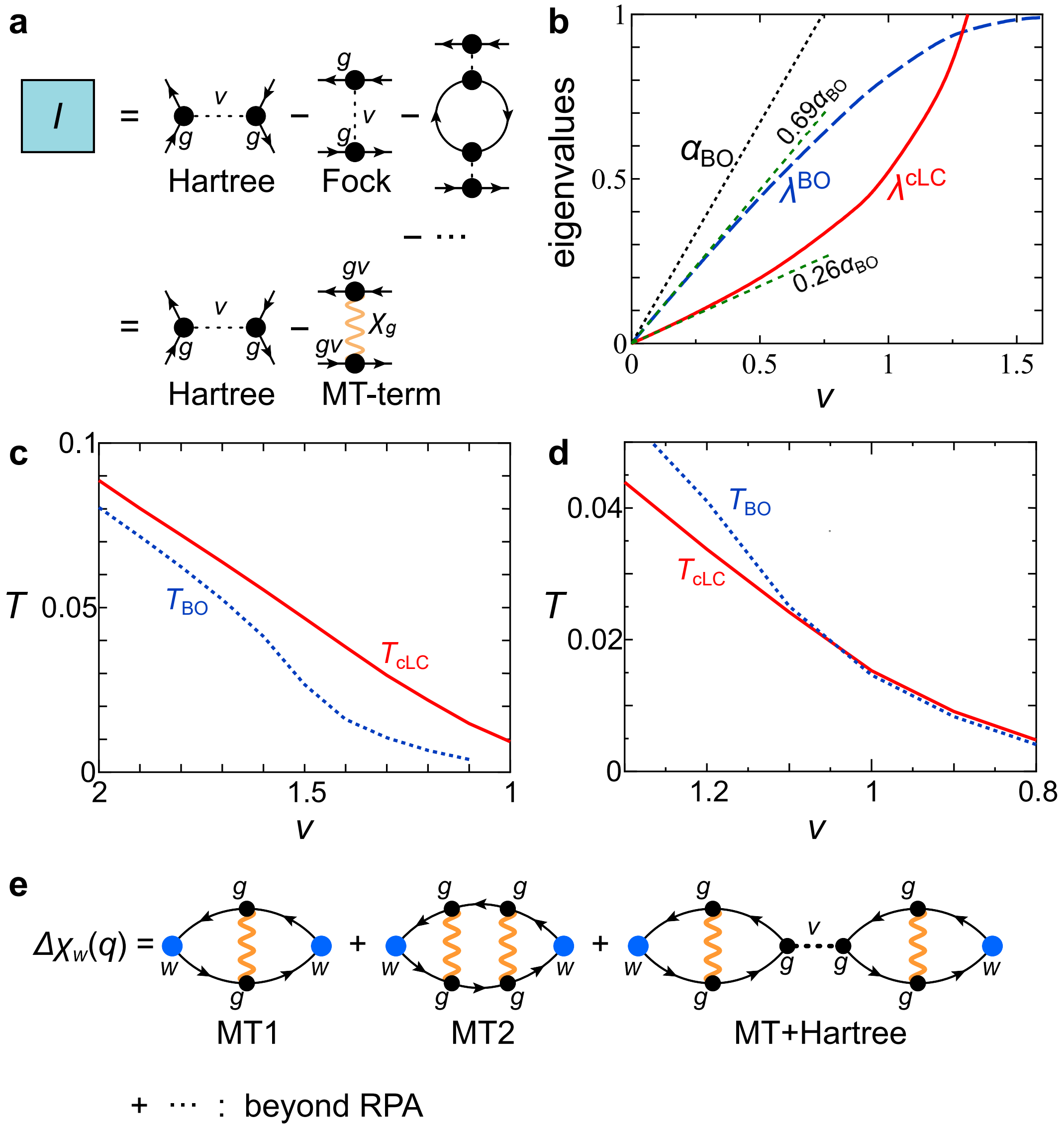

Supplementary Fig. 4: **a**. Diagrammatic expression of the kernel function. **b**. $\lambda_{\rm BO}$, $\lambda_{\rm cLC}$, and $\alpha_{\rm BO}$ given by the present self-consistent density-wave (DW) equation method, for $y = 1$ and $T = 0.01$. **c**.,**d**. Obtained $T_{\rm cLC}$ and $T_{\rm BO}$ as functions of v derived from the self-consistent DW equation method, in the cases of **c** $y = 1.0$ and **d** $y = 0.5$. The self-energy correction is dropped in this calculation. **e**. Beyond-RPA processes $\Delta\chi_w(q)$ generated by solving the DW equation.

In contrast, the AL term is unimportant for the odd-parity order parameter, and instead, the MT term is significant for the current order in the frustrated Hubbard models [7] and non-Fermi liquid transport phenomena [8].

Here, we explain that the AL terms due to the bond susceptibilities, which were neglected in the main text, are unimportant in the present cLC mechanism in kagome metals. Supple-

mentary Figure 5 exhibits the VC for $f^{\rm AB}_{\mathbf{q}_1}(\mathbf{k})$ at $\mathbf{k} \approx \mathbf{k}_{\rm A}$. These terms are almost canceled for the odd-parity cLC order because of the relation $f^{\rm AB}_{\mathbf{q}_1}(\mathbf{k}) = -f^{\rm BA}_{\mathbf{q}_1}(-\mathbf{k}-\mathbf{q})$. In fact, the order parameter in the real space satisfies the relation $\delta t_{ij} = \mathcal{P}\delta t_{ji}$, where $\mathcal{P} = +1$ (-1) for the even (odd) parity order and $l, m = \mathrm{A}, \mathrm{B}, \mathrm{C}$. Then, its Fourier transform gives the form factor:

$$
\begin{aligned}
f^{lm}_{\mathbf{q}}(\mathbf{k}) &= \frac{1}{N}\sum_{i}^{\text{sub}-l}\sum_{j}^{\text{sub}-m} \delta t_{ij} e^{-\mathbf{k}\cdot(\mathbf{r}_i-\mathbf{r}_j)} e^{-\mathbf{q}\cdot\mathbf{r}_j} \\
&= \frac{1}{N}\sum_{i}^{\text{sub}-l}\sum_{j}^{\text{sub}-m} (\mathcal{P}\delta t_{ji}) e^{-\mathbf{k}\cdot(\mathbf{r}_i-\mathbf{r}_j)} e^{-\mathbf{q}\cdot\mathbf{r}_j} \\
&= \mathcal{P} f^{ml}_{\mathbf{q}}(-\mathbf{k}-\mathbf{q}).
\end{aligned} \tag{24}
$$

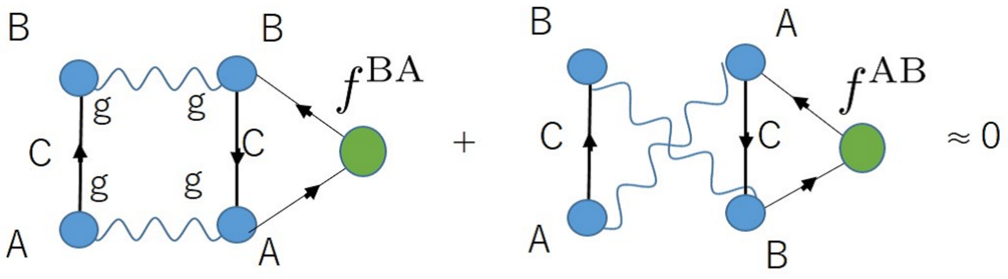

Supplementary Fig. 5: Two Aslamazov-Larkin (AL) terms for the charge loop current (cLC) order at $\mathbf{q} = \mathbf{q}_1$. These terms are almost canceled for the cLC order with $f^{\rm AB}_{\mathbf{q}_1}(\mathbf{k}) = -f^{\rm BA}_{\mathbf{q}_1}(-\mathbf{k}-\mathbf{q}_1)$.

In addition, it is verified that each AL term in Supplementary Fig. 5 is small because the momentum summation is restricted by four g's. (Note that $|g^{lm}_{\mathbf{q}}(\mathbf{k})| \le 1$.)

Supplementary Note 5: Parquet RG theory for kagome metals: Derivation of cLC and BO instabilities

In the main text, we revealed the BO fluctuation-mediated cLC mechanism in kagome metals based on the DW equation method. The BO fluctuations in the MT term of the DW equation causes the scattering between three van-Hove singularity (vHS) points. It is found that the cLC and BO fluctuations develop cooperatively, as demonstrated in Figs. 4 **c-f** in the main text and Supplementary Fig. 4 **b-d**.

The aim of this section is to verify the idea of the BO fluctuation-mediated cLC based on a different reliable theoretical framework. Here, we study the kagome lattice model based on the parquet renormalization group (RG) formulation [13, 14]. Supplementary Figure 6 **a** represents the four scattering processes between three vHS points. g_1 is the backward scattering, g_3 is the Umklapp scattering, and g_2 and g_4 are the forward scatterings. A great merit of the RG method is that both particle-particle and particle-hole channels are treated on the same footing, while the details of the shape of the FS are dropped. Using g_i's, $\Gamma_{\rm um}$ and $\Gamma_{\rm back}$ included in the kernel of the DW equation, which are introduced in the main text, are expressed in Supplementary Fig. 6 **b**. The BO (cLC) instability is given by $\Gamma_{\rm back}+(-)\Gamma_{\rm um}$.

Here, we solve the following parquet RG equation for the three vHS points model to obtain the renormalized g_i ($i=1\sim 4$) due to the electron correlation [13, 14]:

$$\frac{g_1}{dz} = 2dg_1(g_2-g_1), \tag{25}$$

$$\frac{g_2}{dz} = 2d(g_2^2+g_3^2), \tag{26}$$

$$\frac{g_3}{dz} = -g_3^2-2g_3g_4+2dg_3(2g_2-g_1), \tag{27}$$

$$\frac{g_4}{dz} = -2g_3^2-g_4^2, \tag{28}$$

where $z=\Pi^{0,{\rm AAAA}}(\mathbf{0},E)\sim\ln^2(E_0/E)$ and $d=d\chi^{0,{\rm ABBA}}(\mathbf{q}_1,E)/dz$. Here, $\Pi^{0,{\rm AAAA}}(\mathbf{0},E)$ is the Cooper channel bubble with the low-energy (high-energy) cutoff E (E_0), and we consider the effect of the vHS at $E_{\rm F}$. Note that E corresponds to $\sim T$, so $T\approx E_0\exp(-z^{1/2})$. When $E_0=0.2$eV, $z=10$ corresponds to $T\approx 100$K. The parameter d is bounded $0<d<1/2$, and $d=1/2$ corresponds to the perfect nesting. Hereafter, we set $d=1/4$ because the nesting of the real FS is not perfect. In this study, strong cLC and BO instabilities are robustly obtained for $d=1/4\sim 1/2$.

The BO interaction in Eq. (2) in the main text gives the following initial values at $z=0$ (*i.e.*, $E=E_0$): $g_1^0=g_3^0=-v/2$, $g_2^0=0$. They correspond to $y=1/2$ in the main text. Similar sets of $\{g_i^0\}$ were discussed in Ref. [13]. In addition, we include the on-site Coulomb interaction $g_4=U$. Supplementary Figure 6 **c** shows the obtained flows of g_i's as functions of z (≥ 0) for $v=0.6$ and $U=3$. In this case, the renormalized g_1 takes a large negative value, while other g_i's approach zero. In this case, the interaction for the X-channel susceptibility, G_X, is shown in Supplementary Fig. 6 **d**. G_X is given as [13, 14] $G_{\rm cBO}=-2g_1+g_2-g_3$, $G_{\rm cLC}=-2g_1+g_2+g_3$, $G_{\rm sBO}=g_2+g_3$, $G_{\rm sLC}=g_2-g_3$, $G_{s-{\rm SC}}=-2g_3-g_4$, and $G_{d-{\rm SC}}=g_3-g_4$. Here, X =c(s)BO: charge (spin) bond order,

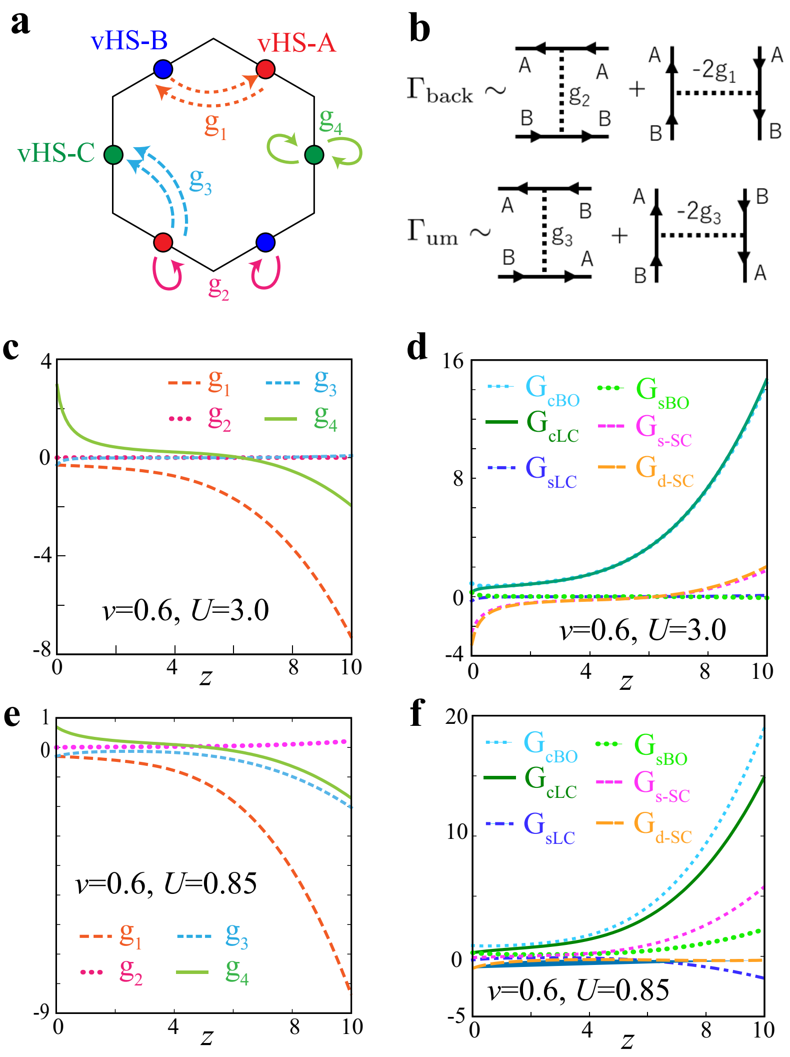

Supplementary Fig. 6: **a**. Scattering processes between three vHS points; g_i ($i = 1 \sim 4$). **b**. Umklapp and backward terms in the kernel of the charge-channel density-wave (DW) equation. $\Gamma_{\rm um} = -g_3$ and $\Gamma_{\rm back} = g_2 - 2g_1$. $\Gamma_{\rm back} + (-)\Gamma_{\rm um}$ gives the cBO (cLC) instability. **c**. Obtained interactions $g_i(z)$ and **d**. instability for the channel X, G_X, (X=cBO, cLC, sBO, sLC, s-SC and d-SC) as function of y, in the case of $v = 0.6$ and $U = 3$. In this case, strong increment of $G_{\rm cBO} \approx G_{\rm cLC}$ are obtained. **e**. Obtained $g_i(z)$ and **f**. G_X in the case of $v = 0.6$ and $U = 0.7$. In this case, the relations $G_{\rm cBO} \gtrsim G_{\rm cLC}$ and $G_{\rm cLC} \sim 2.5 G_{s\rm SC}$ are obtained.

X =c(s)LC: charge (spin) loop-current, $X = s(d)$-SC: $s(d)$-wave SC. Thus, both $G_{\rm cBO}$ and $G_{\rm cLC}$ strongly develop. The relation $G_{\rm cBO} \approx G_{\rm cLC}$ is obtained because g_3 is irrelevant. Therefore, we find $T_{\rm cBC} \approx T_{\rm cLC}$.

Supplementary Figure 6 **e** shows the obtained flow of g_i for $v = 0.6$ and $U = 0.7$. In this

case, both $|g_3|$ and $|g_1|$ are enlarged. ($|g_1| > |g_3|$ is satisfied.) The corresponding instability G_X is shown in Supplementary Fig. 6 **f**. Thus, both $G_{\rm cBO}$ and $G_{\rm cLC}$ are strongly enlarged, while the relation $G_{\rm cBO} \gtrsim G_{\rm cLC} \gtrsim G_{s-{\rm SC}}$ is obtained. Therefore, we find $T_{\rm cBO} \gtrsim T_{\rm cLC} \gtrsim T_{s-{\rm SC}}$.

Note that the CDW instability for the onsite operator $n_{\rm A} + sn_{\rm B}$ ($s = \pm 1$) at $\mathbf{q} = \mathbf{q}_1$ is $G_{{\rm CDW},s} = s(g_1 - 2g_2) - g_4$. Also, the SDW instability for the onsite operator $m_{\rm A} + sm_{\rm B}$ ($m_{\rm A} = n_{{\rm A}\uparrow} - n_{{\rm A}\downarrow}$) at $\mathbf{q} = \mathbf{q}_1$ is $G_{{\rm SDW},s} = sg_1 + g_4$. Both quantities are smaller than $G_{\rm cBO}$ and $G_{\rm cLC}$ after the renormalization, as verified in Supplementary Figs. 6 **d** and **f**.

To summarize, the strong cLC instability derived from the present RG study strongly indicates the validity of the BO fluctuation-mediated cLC mechanism in kagome metals. Considering the initial condition $G^0_{\rm cLC} = G^0_{\rm cBO}/3 = v/2$, the strong cLC instability originates from the beyond-RPA effect. The comparing study between the diagrammatic method and the RG method will lead to further interesting discoveries. On the other hand, parquet RG method cannot derive quantitative results, such as the long-range components of the order parameters obtained from the DW equations.

Supplementary Note 6: Stability of the nematic BO+cLC state

Supplementary Note 6-1: Analytic discussion

In the main text, we explained that the coexistence of the $3Q$ BO and the $3Q$ cLC leads to one C_6 state and three nematic (C_2) states. Here, we discuss the stability of these four states based on the Ginzburg-Landau (GL) theory. It is found that the nematic states are expected to emerge when $T_{\rm BO} > T_{\rm cLC}$ by considering the third-order GL terms, which play essential roles in kagome metals.

Here, we assume that the BO form factor g^{lm} is composed of the nearest-neighbor components, as we did in the main text. Then, the BO form factors $g^{\rm AB}_{\mathbf{q}_1}(\mathbf{k})$, $g^{\rm BC}_{\mathbf{q}_2}$ and $g^{\rm CA}_{\mathbf{q}_3}$ are given by Supplementary Eqs. (1)-(3), respectively. The relation $g^{lm}_{\mathbf{q}}(\mathbf{k}) = \{g^{ml}_{\mathbf{q}}(\mathbf{k})\}^*$ is satisfied. As for the cLC form factor f^{lm}, we use the solution of the DW equation obtained in the main text. The obtained f^{lm} contains long-range components as shown in Fig. 3 **c** in the main text. In fact, its **k**-space expression in Supplementary Figs. 8 **a** and **b** is very different from Supplementary Eq. (1). In addition, the frequency dependence f^{lm} shown

in Supplementary Fig. 8 **c** is very strong, by reflecting drastic frequency dependence of the MT term ($\propto -\chi_g(q)$). The BO [cLC] parameter at $\mathbf{q} = \mathbf{q}_1$ is $\phi_1 g_{\mathbf{q}}^{lm}$ [$\eta_1 f_{\mathbf{q}}^{lm}$] with lm = AB or BA.

Here, we introduce the vector representation $\boldsymbol{\phi} \equiv (\phi_1, \phi_2, \phi_3)$, and define $\boldsymbol{\phi}_1 \equiv (\phi, \phi, \phi)/\sqrt{3}$ and $\boldsymbol{\phi}_2 \equiv (-\phi, \phi, \phi)/\sqrt{3}$. When $\boldsymbol{\phi} = \boldsymbol{\phi}_1$, we obtain the Tri-Hexagonal (Star-of-David) pattern for $\phi > 0$ ($\phi < 0$) shown in Supplementary Fig. 1 **c**. (The free energy for $\boldsymbol{\phi}$ is different from that for $-\boldsymbol{\phi}$ due to the third-order term.) When $\boldsymbol{\phi} = \boldsymbol{\phi}_2$, we obtain the Tri-Hexagonal pattern for $\phi < 0$, while it is displaced by $\mathbf{a}_{\rm AB}$ from Supplementary Fig. 1 **c**.

We also introduce the notation $\boldsymbol{\eta} \equiv (\eta_1, \eta_2, \eta_3)$, and define $\boldsymbol{\eta}_1 \equiv (\eta, \eta, \eta)/\sqrt{3}$ and $\boldsymbol{\eta}_2 \equiv (-\eta, \eta, \eta)/\sqrt{3}$. The cLC pattern in Supplementary Fig. 1 **d** is given by $\boldsymbol{\eta} = \boldsymbol{\eta}_1$ with $\eta > 0$, and the direction of the cLC is reversed when $\eta < 0$. (The free energy is unchanged by $\boldsymbol{\eta} \to -\boldsymbol{\eta}$.) When $\boldsymbol{\eta} = \boldsymbol{\eta}_2$, the cLC pattern is given by the parallel shift of Supplementary Fig. 1 **d** by $\mathbf{a}_{\rm AB}$.

Hereafter, we construct the GL free energy up to the fourth-order terms:

$$F = F^{(2)} + F^{(3)} + F^{(4)}. \tag{29}$$

The second-order term is

$$F^{(2)} = a_1|\boldsymbol{\phi}|^2 + a_2|\boldsymbol{\eta}|^2, \tag{30}$$

$$a_1 = -\chi_g^0(\mathbf{q}_1) + I_{\rm b}^{-1}, \tag{31}$$

$$a_2 = -\chi_f^0(\mathbf{q}_1) + I_{\rm c}^{-1}, \tag{32}$$

where $I_{\rm b(c)}$ (> 0) is the effective interaction for the BO (cLC order) [11, 13]. ($a_{1,2} \le 0$ corresponds to $\lambda_{\mathbf{q}} \ge 1$ in the DW equation as proved in Ref. [11].) Because $\chi_g^0(\mathbf{q}_1) \approx \chi_f^0(\mathbf{q}_1)$, the relation $T_{\rm BO} > T_{\rm cLC}$ would be realized when $I_{\rm b} > I_{\rm c}$. We note that, in the present theory, both $I_{\rm b}$ and $I_{\rm c}$ originate from the electron correlations, and therefore they exhibit strong T-dependences.

The third-order term and the forth-order term for general $\boldsymbol{\phi}$ and $\boldsymbol{\eta}$ are given as

$$F^{(3)} = b_1\phi_1\phi_2\phi_3 + b_2(\phi_1\eta_2\eta_3 + \eta_1\phi_2\eta_3 + \eta_1\eta_2\phi_3), \tag{33}$$

$$\begin{aligned} F^{(4)} = &\ d_{1,a}(\phi_1^4 + \phi_2^4 + \phi_3^4) + d_{1,b}(\phi_1^2\phi_2^2 + \phi_2^2\phi_3^2 + \phi_3^2\phi_1^2) \\ &+ d_{2,a}(\eta_1^4 + \eta_2^4 + \eta_3^4) + d_{2,b}(\eta_1^2\eta_2^2 + \eta_2^2\eta_3^2 + \eta_3^2\eta_1^2) \\ &+ 2d_{3,a}(\phi_1^2\eta_1^2 + \phi_2^2\eta_2^2 + \phi_3^2\eta_3^2) \end{aligned}$$

$$+d_{3,b}(\phi_1^2\eta_2^2 + \phi_2^2\eta_3^2 + \phi_3^2\eta_1^2 + \phi_2^2\eta_1^2 + \phi_3^2\eta_2^2 + \phi_1^2\eta_3^2). \tag{34}$$

Note that the term $\sim \phi_1\phi_2\eta_1\eta_2$ is absent. In the absence of the cLC order, the third-order free energy term is given by the first term of Supplementary Eq. (33). The diagrammatic expression for the coefficient b_1 is given in Supplementary Fig. 7 **a**, and its analytic expression is found in Refs. [12, 13]. When the BO and the cLC coexist, we obtain the additional cross-terms given by the second term of Supplementary Eq. (33). The diagrammatic expression for b_2 is given in Supplementary Fig. 7 **b**. The relation $b_1 = -b_2$ holds when the relation $f^{\rm BC}_{\mathbf{q}_2}(\mathbf{k}_{\rm C}) f^{\rm CA}_{\mathbf{q}_3}(\mathbf{k}_{\rm A}) = -g^{\rm BC}_{\mathbf{q}_2}(\mathbf{k}_{\rm C}) g^{\rm CA}_{\mathbf{q}_3}(\mathbf{k}_{\rm A})$ holds. Note that the minimum of the GL free energy does not diverge due to the positive fourth-order term.

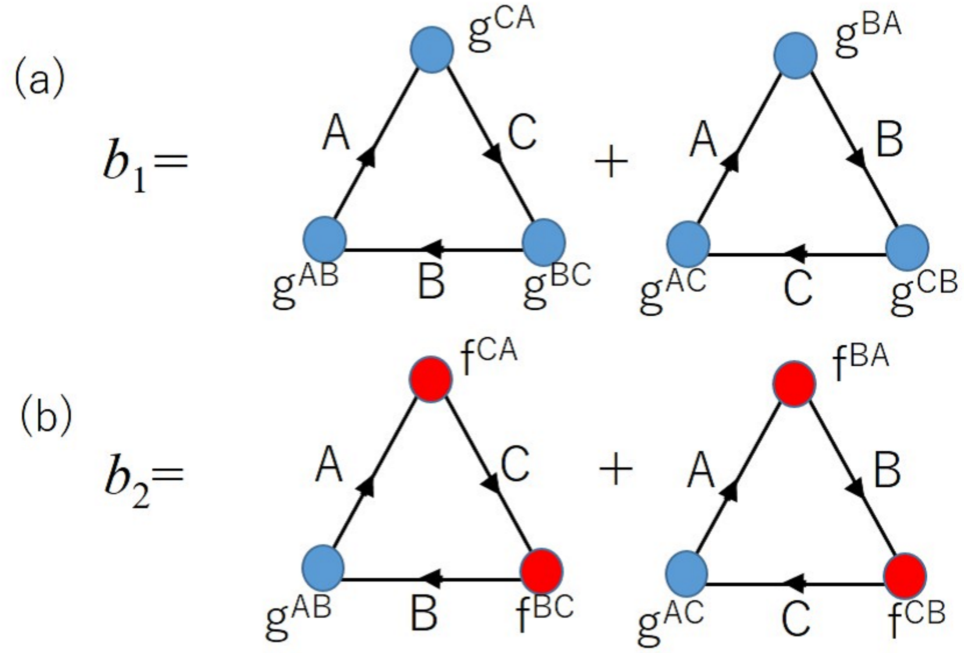

Supplementary Fig. 7: Diagrams for the third-order Ginzburg-Landau terms b_1 and b_2. The relation $b_1 = -b_2$ holds when $f^{lm}_{\mathbf{q}}(\mathbf{k}) = \pm i g^{lm}_{\mathbf{q}}(\mathbf{k})$.

Now, we set $\boldsymbol{\phi} = \boldsymbol{\phi}_1$ because the $3Q$ BO is stabilized by the third-order GL free energy ($\propto \phi_1\phi_2\phi_3$), and it is actually observed experimentally. When $\boldsymbol{\eta} = \boldsymbol{\eta}_{1[2]}$, the BO+cLC state is C_6 $[C_2]$ symmetric as shown in Fig. 5 **a** [**b**] in the main text. Hereafter, we explain that the C_2-symmetric BO+cLC state is realized due to $\boldsymbol{\eta} \neq \boldsymbol{\eta}_1$, based on both analytic and numerical studies.

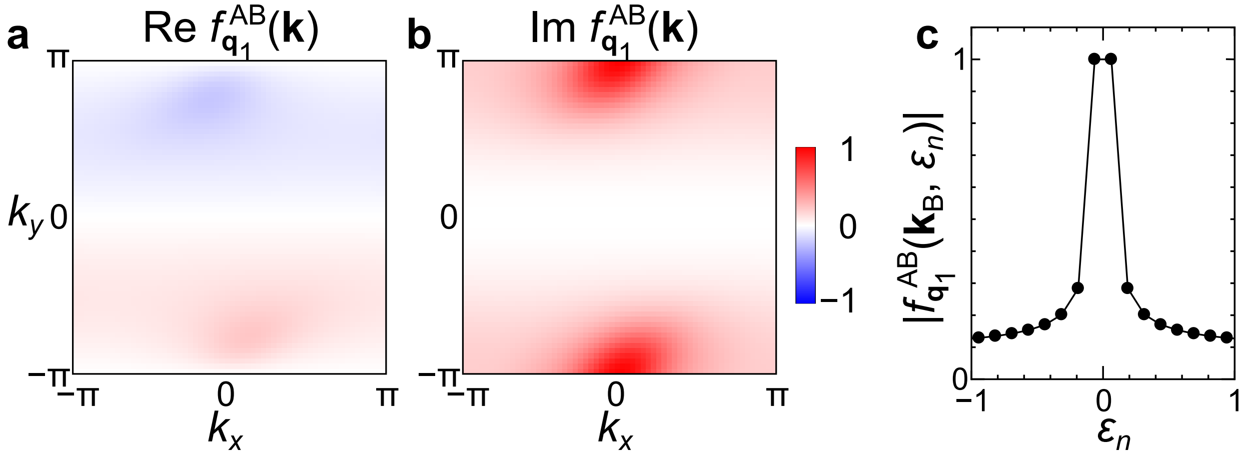

Supplementary Fig. 8: **a**. Real part and **b**. imaginary part of the cLC form factor $f^{\rm AB}_{\mathbf{q}_1}(\mathbf{k})$ in the square kagome-lattice model. $f^{\rm AB}_{\mathbf{q}_1}(\mathbf{k})$ is very different from the nearest-neighbor BO form factor $-ig^{\rm AB}_{\mathbf{q}_1}(\mathbf{k}) = \frac{1}{2}\sin k_y - i\frac{1}{2}(1-\cos k_y)$, and its strong $\mathbf{k}$-dependence means the existence of large cLC order between distant sites. This means that the inner-product $|\frac{1}{N}\sum_{\mathbf{k}}[if^{\rm AB}_{\mathbf{q}}(\mathbf{k})]^* g^{\rm AB}_{\mathbf{q}}(\mathbf{k})|$ is small, indicating that the cLC and the BO are nearly independent. **c**. ϵ_n-dependence of $|f^{\rm AB}_{\mathbf{q}_1}(\mathbf{k}_B, e_n)|$, where $y = 1$, $v = 1.0$ and $T = 0.02$.

Here, we discuss the possible $3Q$ BO+cLC states in the case of $T_{\rm BO} > T_{\rm cLC}$. When $\boldsymbol{\phi} = \boldsymbol{\phi}_1$, the third-order term is

$$F^{(3)'} = \frac{b_1}{3\sqrt{3}}\phi^3 + \frac{b_2}{2\sqrt{3}}\phi[(\eta_1+\eta_2+\eta_3)^2 - |\boldsymbol{\eta}|^2]. \quad (35)$$

Now, we minimize Supplementary Eq. (35) under the constraints $|\boldsymbol{\eta}|$ =const. and $|\boldsymbol{\phi}|$ =const., where the 2nd order GL free energy is constant. In the case of $|\boldsymbol{\eta}| \gg |\boldsymbol{\phi}|$ ($T_{\rm cLC} \gg T_{\rm BO}$), $b_2\phi$ becomes negative and $\boldsymbol{\eta} = \boldsymbol{\eta}_1$ to get the energy gain from the second term in Supplementary Eq. (35). (The first term is small and positive because of $b_1 b_2 < 0$.) Thus, the coexisting state has C_6 symmetry.

In the case of $|\boldsymbol{\eta}| \ll |\boldsymbol{\phi}|$ ($T_{\rm cLC} \ll T_{\rm BO}$), $b_1\phi$ becomes negative to get the energy gain from the first term in Supplementary Eq. (35). Then, the second term is minimized when $\eta_1+\eta_2+\eta_3 = 0$ for a fixed $|\boldsymbol{\eta}|$. For example, $\boldsymbol{\eta}' \propto (\eta, \eta, -2\eta)$ or $\boldsymbol{\eta}'' \propto (\eta, -\eta, 0)$. In both cases, the coexisting state has C_2 symmetry. The nematic BO+cLC state with $\boldsymbol{\phi}_1$ and $\boldsymbol{\eta}'$ is depicted in Fig. 5 **c** in the main text. Note that the total free energy is unchanged under $\eta_1+\eta_2+\eta_3 = 0$ within the 4th-order GL terms, while this degeneracy is lifted by the

6th-order GL terms. Thus, the Z_3 nematic BO+cLC state by $\boldsymbol{\eta}'$ or $\boldsymbol{\eta}''$ is expected to be realized for $T < T_{\rm cLC} < T_{\rm BO}$, as shown in a schematic phase diagram in Fig. 4 **f** in the main text.

Finally, we discuss the fourth-order GL term in Supplementary Eq. (34). The coefficients $d_{l,a}$ and $d_{l,b}$ ($l = 1, 2, 3$) are positive, and they are given by the closed Feynman diagrams made of four form factors and four G's [13]. The relations $d_{1,a} = d_{2,a} = d_{3,a}$ and $d_{1,b} = d_{2,b} = d_{3,b}$ hold in the case of $f^{lm}_{\mathbf{q}}(\mathbf{k}) \approx \pm i g^{lm}_{\mathbf{q}}(\mathbf{k})$. For any $\boldsymbol{\phi} = \boldsymbol{\phi}_\alpha$ and $\boldsymbol{\eta} = \boldsymbol{\eta}_\beta$ ($\alpha, \beta = 1, 2$), the fourth-order GL term is expressed as

$$F^{(4)'} = d_1\phi^4 + d_2\eta^4 + 2d_3\phi^2\eta^2, \tag{36}$$

where the coefficients are $d_1 = (d_{1,a} + d_{1,b})/3$, $d_2 = (d_{2,a} + d_{2,b})/3$, and $d_3 = (d_{3,a} + d_{3,b})/3$. However, the relation $f^{lm}_{\mathbf{q}}(\mathbf{k}) \approx \pm i g^{lm}_{\mathbf{q}}(\mathbf{k})$ is not satisfied because the mechanisms of the cLC and the BO are different in the present theory. Therefore, all the coefficients b_l, $d_{m,a}$, $d_{m,b}$ depend on $l = 1, 2$ and $m = 1, 2, 3$. In the successive section, we discuss the stability of the nematic BO+cLC state based on the numerical study.

Supplementary Note 6-2: Numerical study

Here, we calculate the 3rd- and 4th-order GL coefficients based on the diagrammatic method. We normalize the form factors as $\max_{\mathbf{k}} |g^{\rm AB}_{\mathbf{q}_1}(\mathbf{k})| = \max_{\mathbf{k},l,m} |f^{\rm AB}_{\mathbf{q}_1}(\mathbf{k})| = 1$ which is realized at $\mathbf{k} = \mathbf{k}_{\rm A}$ in the present DW equation analysis. Then, the BO parameter $\phi_m g_{\mathbf{q}_m}(\mathbf{k})$ at $\mathbf{q} = \mathbf{q}_m$ ($m = 1 \sim 3$) gives the hybridization gap $\Delta_{\rm BO} \sim |\phi|$ in the folded band at Γ point. [In the same way, $\Delta_{\rm cLC} \sim |\eta|$ for the cLC order $\eta_m f_{\mathbf{q}_m}(\mathbf{k})$.] Thus, the present normalization rule for g and f is physically reasonable and convenient. Note that the normalization rule does not influence the physical quantities, because the change of $H_{\rm int}$ due to $g \to r \cdot g$ is absorbed by $v \to v/r^2$.

The 3rd order GL parameters per unit cell are given as

$$b_1 = 3I^{ggg}_{123} + 3I^{ggg}_{132}, \tag{37}$$

$$b_2 = 3I^{gff}_{123} + 3I^{gff}_{132}, \tag{38}$$

where

$$I^{xyz}_{lmn} = -\frac{T}{3N}\sum_{k,\sigma} {\rm Tr}\hat{x}_{q_l}(k + q_n + q_m)\hat{G}(k + q_n + q_m)$$

$$\times \; \hat{y}_{q_m}(k+q_n)\hat{G}(k+q_n)\hat{z}_{q_n}(k)\hat{G}(k), \tag{39}$$

where x, y, z is f or g, and l, m, n is 1, 2, or 3. The relation $q_l + q_m + q_n = 0$ should be satisfied. Here, $\hat{G}(k)$ is the 3×3 matrix expression of the Green function with the self-energy given in the main text.

The 4th order GL parameters per unit cell are given as

$$d_{1,a} = I^{gggg}_{1111}, \tag{40}$$

$$d_{1,b} = 2I^{gggg}_{1212} + 4I^{gggg}_{1122}, \tag{41}$$

$$d_{2,a} = I^{ffff}_{1111}, \tag{42}$$

$$d_{2,b} = 2I^{ffff}_{1212} + 4I^{ffff}_{1122}, \tag{43}$$

$$d_{3,a} = \frac{1}{2}(2I^{fgfg}_{1111} + 4I^{ffgg}_{1111}), \tag{44}$$

$$d_{3,b} = 2I^{fgfg}_{1212} + 4I^{ffgg}_{1122}, \tag{45}$$

where

$$\begin{aligned} I^{wxyz}_{hlmn} &= \frac{T}{4N}\sum_{k,\sigma} \mathrm{Tr}\hat{w}_{q_h}(k+q_n+q_m+q_l)\hat{G}(k+q_n+q_m+q_l) \\ &\times \; \hat{x}_{q_l}(k+q_n+q_m)\hat{G}(k+q_n+q_m) \\ &\times \; \hat{y}_{q_m}(k+q_n)\hat{G}(k+q_n)\hat{z}_{q_n}(k)\hat{G}(k), \end{aligned} \tag{46}$$

where w, x, y, z is f or g, and h, l, m, n is 1, 2, or 3. The relation $q_h + q_l + q_m + q_n = 0$ should be satisfied. The diagrammatic expression of I^{xyz}_{lmn} and I^{wxyz}_{hlmn} are depicted in Supplementary Fig. 9.

Note that these GL coefficients depend on the Green functions (=bandstructure) and the form factors g and f. Thus, the GL coefficients depend on the BO/cLC mechanisms indirectly, just through the form factors.

The numerical results of the 3rd and 4th GL parameters are shown in Supplementary Figs. 10 **a** and **b**, respectively, for $v = 0.6$ and $y = 1$. We find that b_1 and b_2 have different signs, consistently with the discussion in the previous section. The obtained GL coefficients exhibit moderate and monotonic T-dependences for a wide T range because the self-energy suppresses unrealistic singular behaviors of GL coefficients at low temperatures. As we show in Supplementary Fig. 10 **c**, the obtained ratio $r = 2d_{1,a}/d_{1,b}$ is larger than 1. This result means that experimentally observed $3Q$ BO state is realized irrespective of the size of b_1. Also, the relation $r' = 2d_{2,a}/d_{2,b} > 1$ means that the $3Q$ cLC state is realized.

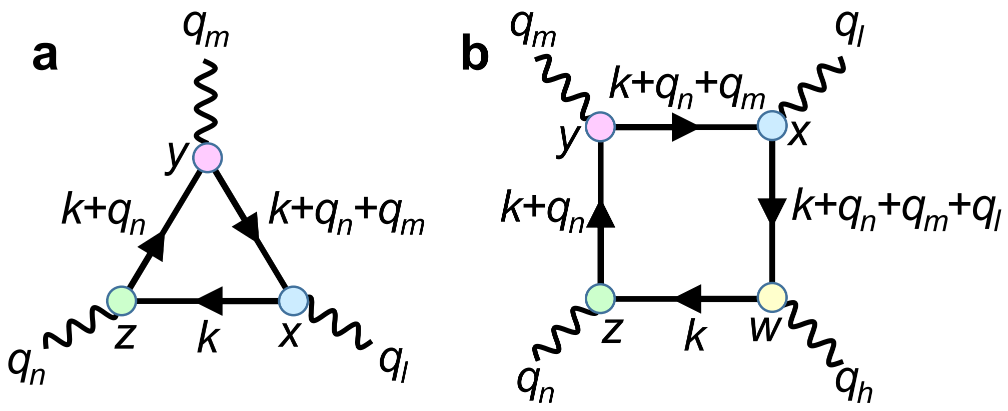

Supplementary Fig. 9: Diagrammatic expressions of Ginzburg-Landau parameters: **a**. Third-order term. **b**. Fourth-order term.

In addition, the obtained GL coefficients satisfy the relations $R = d_{1,a}d_{2,a}/d_{3,a}^2 > 1$ and $R' = d_{1,b}d_{2,b}/d_{3,b}^2 > 1$ as shown in Supplementary Fig. 10 **d**. These relations indicate the smallness of the competition between cLC and BO described by $d_{3,a}$ and $d_{3,b}$ terms. (Note that $R = R' = 1$ when the BO and cLC form factors are composed of only the nearest bonds [13].) Therefore, we can expect the coexistence of the BO and cLC orders by analyzing the GL free energy. The relations $R, R' > 1$ originates from the smallness of the inner-product $|\frac{1}{N}\sum_{\mathbf{k}}[if_{\mathbf{q}}^{ml}(\mathbf{k})]^* g_{\mathbf{q}}^{lm}(\mathbf{k})|$ (see Supplementary Fig. 8 **a** and **b**) and the strong frequency dependence of the cLC form factor shown in Supplementary Fig. 8 **c**. This is not surprising because the driving forces of the BO and the cLC are different in the present theory.

Supplementary Figure 11 **a** exhibits the obtained phase diagram derived from the GL free energy, by using the GL coefficients in Supplementary Figs. 10 **a** and **b** at $T = 0.01$. The horizontal (vertical) axis is the second-order GL parameter for the BO a_1 (cLC a_2). Each phase is determined by minimizing the GL free energy $F[\boldsymbol{\phi}, \boldsymbol{\eta}]$ with respect to the following 13^2 patterns $(\boldsymbol{\phi}, \boldsymbol{\eta}) = (\boldsymbol{\phi}_m, \boldsymbol{\eta}_n)$ with $m, n = 1 \sim 13$ exactly numerically:

$$
\begin{aligned}
3Q \text{ BO}: \quad & \boldsymbol{\phi}_1 = \phi(1,1,1)/\sqrt{3}, \\
& \boldsymbol{\phi}_2 = \phi(-1,1,1)/\sqrt{3}, \quad \boldsymbol{\phi}_3,\ \boldsymbol{\phi}_4 = \text{cycl.}, \\
2Q \text{ BO}: \quad & \boldsymbol{\phi}_5 = \phi(1,1,0)/\sqrt{2}, \quad \boldsymbol{\phi}_6,\ \boldsymbol{\phi}_7 = \text{cycl.}, \\
& \boldsymbol{\phi}_8 = \phi(1,-1,0)/\sqrt{2}, \quad \boldsymbol{\phi}_9,\ \boldsymbol{\phi}_{10} = \text{cycl.}, \\
1Q \text{ BO}: \quad & \boldsymbol{\phi}_{11} = \phi(1,0,0), \quad \boldsymbol{\phi}_{12},\ \boldsymbol{\phi}_{13} = \text{cycl.},
\end{aligned}
$$

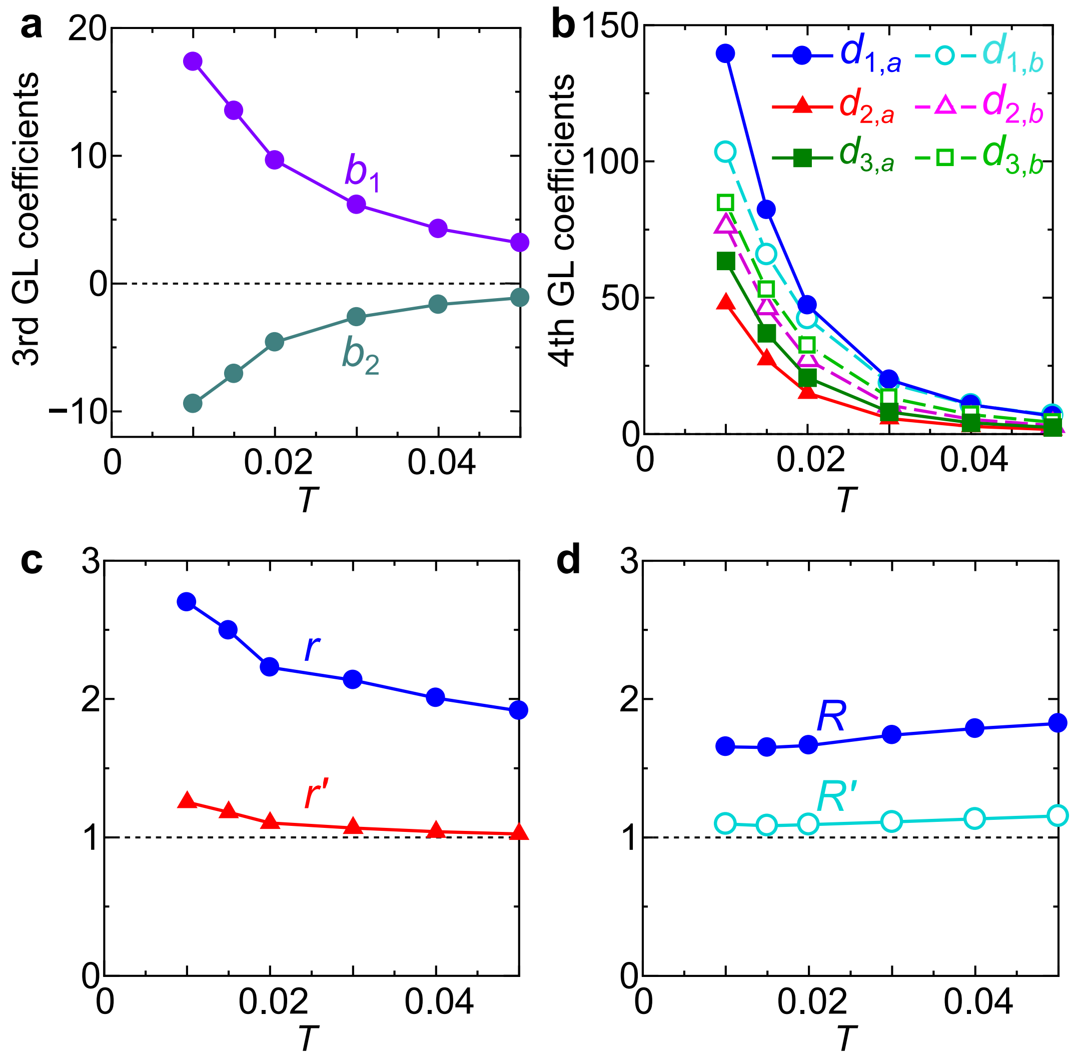

Supplementary Fig. 10: **a.**,**b**. Numerical results of the Ginzburg-Landau parameters for $v = 0.6$ as functions of T. The relation $b_1 b_2 < 0$ is verified. **c**. Ratio $r = 2d_{1,a}/d_{1,b}$ (for BO) and ratio $r' = 2d_{2,a}/d_{2,b}$ (for cLC). The $3Q$ state is stable when r (r') is larger than unity. **d**. Ratio $R = d_{1,a}d_{2,a}/d_{3,a}^2$ and ratio $R' = d_{1,b}d_{2,b}/d_{3,b}^2$. The C_2 symmetric BO+cLC coexisting state is energetically stable when $R, R' > 1$.

and $\boldsymbol{\eta}_n = \boldsymbol{\phi}_n|_{\phi\to\eta}$ for the cLC.

The obtained C_2 symmetric BO+cLC phase with $\boldsymbol{\phi} = \boldsymbol{\phi}_1$ and $\boldsymbol{\eta} = (\eta, -\eta, 0)/\sqrt{2}$ (or equivalently $\boldsymbol{\eta} = (\eta, \eta, -2\eta)/\sqrt{6}$; see E-1) is shown by the purple region. This C_2 coexisting phase is always realized when $T_{\rm BO} \gtrsim T_{\rm cLC}$. Also, the green (red) region corresponds to $3Q$ BO (cLC) phase. In the red region, the secondary $3Q$ BO appears through the b_2-terms even for $a_1 > 0$, and $|\boldsymbol{\phi}|$ becomes comparable to $|\boldsymbol{\eta}|$ when $a_1 < 0$. The coexisting state is C_6

symmetry. [13].

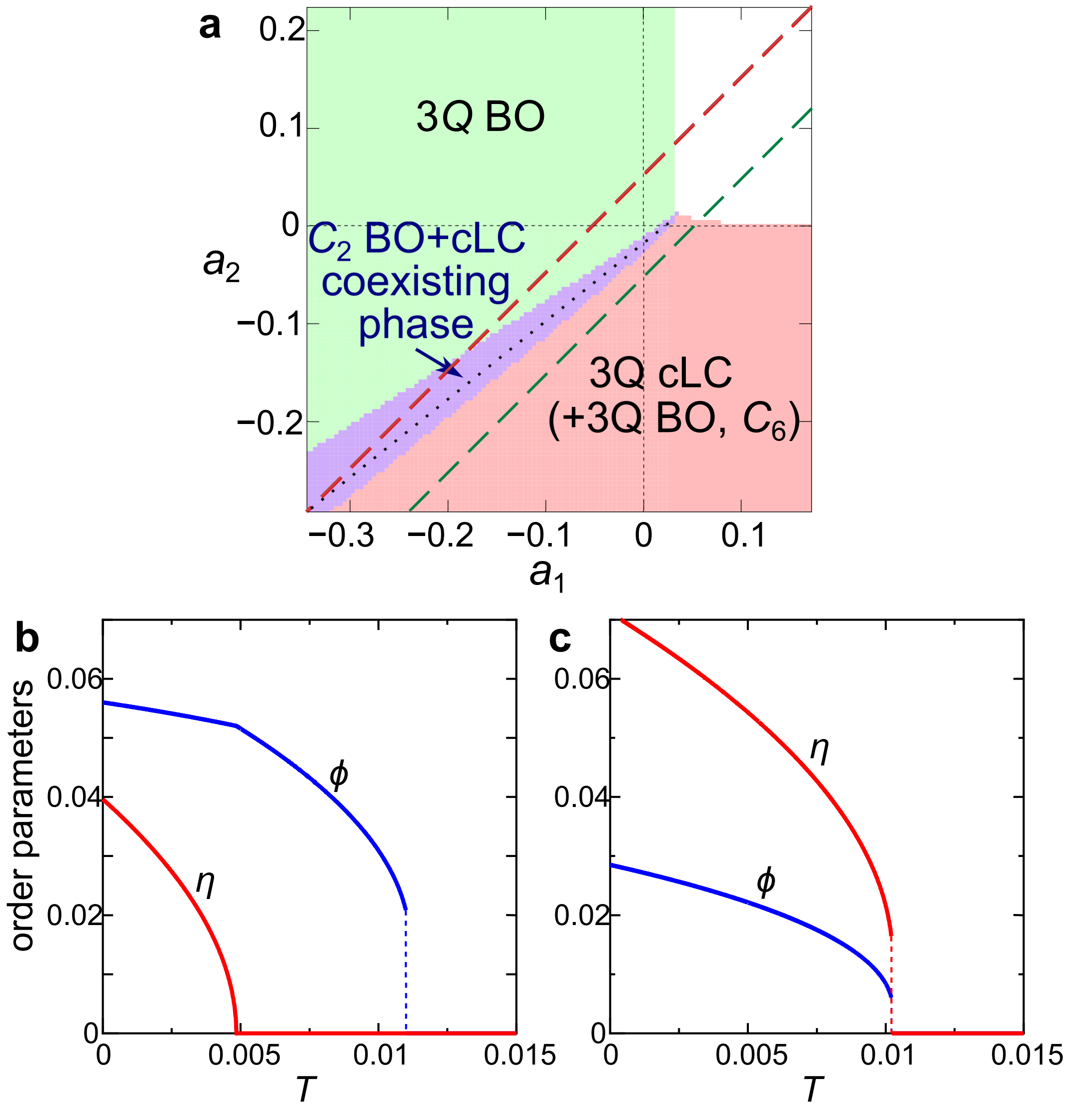

Supplementary Fig. 11: **a**. Obtained phase diagram using the Ginzburg-Landau coefficients in Supplementary Figs. 10 **a** and **b** at $T = 0.01$. The horizontal (vertical) axis is a_1 (a_2). The purple region represents the C_2 symmetric BO+cLC phase. The dotted line is the phase boundary without optimizing the C_2 BO and cLC order parameters. **b**.,**c**. Obtained order parameters along **b** the red broken line and **c** the green broken line in **a**.

In deriving the phase boundary between the C_2 and C_6 coexisting phases in Supplementary Fig. 11 **a**, we optimized three parameters $\{\phi, \phi', \eta\}$ numerically by considering additional 14th order parameter $\boldsymbol{\phi}_{14} \sim (\phi, \phi, \phi')$ and $\boldsymbol{\eta} \sim (\eta, -\eta, 0)$. The dotted line in Supplementary Fig. 11 **a** is the phase boundary by setting $\phi = \phi'$.

Supplementary Figure 11 **b** shows the obtained order parameters along the red broken line in **a**. Here, we set 2nd order GL parameters as functions of T: $a_1 = A_1(T/T^0_{\rm BO} - 1)$, $a_2 = A_2(T/T^0_{\rm cLC} - 1)$, where $A_1 = A_2 = 0.17$, $T^0_{\rm BO} = 0.01$ and $T^0_{\rm cLC} = 0.85T^0_{\rm BO}$. Here, $A_{1(2)} = 0$ at $T = T^0_{\rm BO(cLC)}$. The GL parameters at $T = 0.01$ in Supplementary Fig. 10 are used. We find that $|\boldsymbol{\phi}|$ exhibits the 1st order transition at $T_{\rm BO} \approx 1.1T^0_{\rm BO}$, and $|\boldsymbol{\eta}|$ appears as the 2nd-order transition at $T_{\rm cLC} \approx 0.6T^0_{\rm cLC} \approx 0.5T^0_{\rm BO}$. This result corresponds to the region $v > v^*$ in Fig. 4 **f** in the main text. By performing careful numerical analyses, we find that the C_2 coexisting region (=purple region in Supplementary Figure 11 **a**) appears when the relations $R > 1$ and $R' > 1$ are satisfied.

Supplementary Figure 11 **c** shows the order parameters along the green broken line in **a**. The primary $3Q$ cLC order induces the secondary $3Q$ BO due to the 3rd order GL terms. The symmetry of this coexisting phase is C_6 [13].

---

* Electronic address: `rina.tazai@yukawa.kyoto-u.ac.jp`

[1] R. Tazai, Y. Yamakawa, S. Onari, and H. Kontani, *Mechanism of exotic density-wave and beyond-Migdal unconventional superconductivity in kagome metal AV_3Sb_5 (A = K, Rb, Cs)*, Sci. Adv. **8**, eabl4108 (2022).

[2] S. Onari and H. Kontani, *Self-consistent Vertex Correction Analysis for Iron-based Superconductors: Mechanism of Coulomb Interaction-Driven Orbital Fluctuations*, Phys. Rev. Lett. **109**, 137001 (2012).

[3] Y. Yamakawa, S. Onari, and H. Kontani, *Nematicity and Magnetism in FeSe and Other Families of Fe-Based Superconductors*, Phys. Rev. X **6**, 021032 (2016).

[4] S. Onari, Y. Yamakawa, and H. Kontani, *Sign-Reversing Orbital Polarization in the Nematic Phase of FeSe due to the C_2 Symmetry Breaking in the Self-Energy*, Phys. Rev. Lett. **116**, 227001 (2016).

[5] M. Tsuchiizu, K. Kawaguchi, Y. Yamakawa, and H. Kontani, *Multistage electronic nematic transitions in cuprate superconductors: A functional-renormalization-group analysis*, Phys. Rev. B **97**, 165131 (2018).

[6] Y. Yamakawa and H. Kontani, *Spin-Fluctuation-Driven Nematic Charge-Density Wave in Cuprate Superconductors: Impact of Aslamazov-Larkin Vertex Corrections*, Phys. Rev. Lett.

114, 257001 (2015).

[7] R. Tazai, Y. Yamakawa, and H. Kontani, *Emergence of charge loop current in the geometrically frustrated Hubbard model: A functional renormalization group study*, Phys. Rev. B **103**, L161112 (2021).

[8] H. Kontani, *Anomalous transport phenomena in Fermi liquids with strong magnetic fluctuations*, Rep. Prog. Phys. **71**, 026501 (2008).

[9] H. Kontani, R. Tazai, Y. Yamakawa, and S. Onari, *Unconventional density waves and superconductivities in Fe-based superconductors and other strongly correlated electron systems*, Adv. Phys. **70**, 355 (2021).

[10] T. Moriya and K. Ueda, *Spin fluctuations and high temperature superconductivity*, Adv. Phys. **49**, 555 (2000).

[11] R. Tazai, S. Matsubara, Y. Yamakawa, S. Onari, and H. Kontani, *A Rigorous Formalism of Unconventional Symmetry Breaking in Fermi Liquid Theory and Its Application to Nematicity in FeSe*, Phys. Rev. B **107**, 035137 (2023).

[12] T. Hirata, Y. Yamakawa, S. Onari, and H. Kontani, *Unconventional orbital charge density wave mechanism in the transition metal dichalcogenide* $1T-TaS_2$, Phys. Rev. Research **3**, L032053 (2021).

[13] T. Park, M. Ye, and L. Balents, *Electronic instabilities of kagome metals: Saddle points and Landau theory*, Phys. Rev. B **104**, 035142 (2021).

[14] R. Nandkishore, L. S. Levitov, and A. V. Chubukov, *Chiral superconductivity from repulsive interactions in doped graphene*, Nat. Phys. **8**, 158 (2012).
